# Design and Validation of a Pressure-Driven Liquid Metering System with Heated PTFE Tubing for Laboratory Automation

**DOI:** 10.3390/s26020700

**Published:** 2026-01-21

**Authors:** Joonki Baek, Taegyun Kim, Seungwon Jeong, Ikhyun Kim, Shin Hum Cho, Sungkeun Yoo

**Affiliations:** 1 Department of Robot Engineering, Keimyung University, 1095 Dalgubeol-daero, Daegu 42601, Republic of Korea; 1114830@stu.kmu.ac.kr (J.B.); 5588874@stu.kmu.ac.kr (S.J.); 2 Department of Mechanical Engineering, BK21 FOUR ERICA-ACE Center, Hanyang University, 55 Hanyangdaehak-ro, Ansan 15588, Republic of Korea; taegyunkim@hanyang.ac.kr; 3 Department of Mechanical Engineering, Keimyung University, 1095 Dalgubeol-daero, Daegu 42601, Republic of Korea; kimih@kmu.ac.kr; 4 Department of Chemical Engineering, Keimyung University, 1095 Dalgubeol-daero, Daegu 42601, Republic of Korea; shinhum@kmu.ac.kr

**Keywords:** laboratory automation, pressure-driven dispensing, precision liquid metering, temperature-dependent viscosity

## Abstract

This paper presents a pressure-driven liquid transfer system for laboratory automation, along with a physics-based model and calibration method. The device maintains near-isothermal transport by storing reagents at a prescribed temperature and routing the flow through a single PTFE tube enclosed within a heated jacket. The pressure-drop model accounts for temperature-dependent viscosity and the thermal expansion of PTFE. Residual deviations from the no-slip prediction in submillimeter tubing are represented by an effective slip length, which is identified through linear regression. This parameter is subsequently used to calculate the pressure required to achieve a target flow rate. Experimental results compare unheated and heated operating conditions and characterize the dependence of slip length on temperature and flow rate. Under heated operation with slip-compensated pressure commands, the system achieved dispensing accuracy within ±4% over the tested range, whereas unheated operation exhibited larger errors due to axial temperature gradients. These results demonstrate that effective thermal management and slip compensation are critical for accurate pressure-based metering under temperature-sensitive conditions, as validated using water-based tests.

## 1. Introduction

Laboratory chemistry and biology rely heavily on manual, experiment-intensive procedures. Even small handling errors can degrade experimental outcomes, and as a result, complex protocols are often executed by the scientists who design them. As industrial research scales, operators are increasingly required to handle hazardous materials and follow intricate procedures with tight tolerances. This trend elevates safety risks, complicates accurate execution, and reduces reproducibility. ISO 8655 specifies standardized procedures for testing pipette accuracy and precision, including the single-channel air-displacement test cycle [1]. However, in practice, operator skill introduces substantial variability. In a cohort of ten novice operators, many measurements exceeded specified tolerances across different liquids and volumes, with the highest failure rate observed for water at volumes of 20 μL or less [2].

To mitigate human-induced variability, automated pipetting systems are increasingly adopted. A 3D-printed digital pipette mounted on a robotic manipulator achieved volumetric errors of approximately 0.5% or lower while closely adhering to ISO 8655 evaluation procedures [3]. As demand for automated liquid handling (ALH) grows, low-cost approaches have expanded access beyond proprietary pipetting heads. For example, converting hobbyist 3D printers into liquid handlers and integrating manual pipettes has yielded practical accuracy at entry costs of approximately USD 300–400 [4,5]. In parallel, open-source benchtop platforms such as the Opentrons OT-2 have supported reproducible RT-qPCR COVID-19 testing workflows, demonstrating end-to-end protocol automation on commodity hardware [6,7]. New open-hardware designs (e.g., BioCloneBot) further illustrate how syringe-pump or pipette-based architectures can deliver usable precision for routine laboratory tasks at low cost [8]. Another compact, low-cost device designed for simplicity, scalability, and personalization, PHIL, enables automated pipetting directly on microscope stages. At higher dispensing speeds, it exhibits approximately a 5% deviation, indicating that further calibration can improve accuracy [9]. In addition to DIY platforms, commercial pipetting systems (e.g., the TECAN Genesis/Freedom EVO series) also require rigorous gravimetric calibration. Balance-based procedures are standard practice and have been shown to verify precision and improve accuracy on these platforms [10,11].

For continuous metering applications, syringe pumps and various microfluidic pump architectures are widely used [12,13,14]. Syringe pumps dispense fluid by commanding plunger displacement, such that the delivered volume is nominally proportional to plunger motion [15,16]. Stepper motor control enables low flow rates, and valve-equipped systems can alternate between aspiration and dispensing when throughput exceeds syringe capacity [17,18]. In practice, however, mechanical compliance, stiction, and friction within the plunger–barrel interface delay pressure buildup, meaning that motor rotation does not immediately generate flow [19]. After pressurization, motor vibrations and stick–slip behavior can induce flow ripple, an effect that typically intensifies at higher flow rates [19,20,21]. When dispensing high-viscosity liquids, viscous losses can drive pressures high enough to immobilize the plunger or damage the syringe.

Pressure-driven metering using proportional control valves (PCVs) transmits actuator pressure to the working fluid with minimal compliance, enabling fast dynamics and making this approach attractive for periodic or pulsatile delivery [22]. Closed-loop implementations that weigh dispensed mass using load cells can improve accuracy [23], and balance-based instruments can achieve high resolution. Coriolis-based feedback control has demonstrated stable delivery at $\;100\;\mathrm{mg}\;\min^{-1}$ with errors within ±1% [24]. In that implementation, the high accuracy primarily reflected reliance on the sensor’s characteristics, particularly its relative insensitivity to temperature, density, and viscosity. Using a simple first-order model identified from data, a PI closed loop regulating headspace pressure achieved a coefficient of variation (CV) below 3% and recovered from a 10 mbar disturbance in 0.3 s [25]. These results indicate that sensor-feedback pressure control can meet target accuracy within the sensor’s measurement envelope even with basic controllers, and that physics-based system modeling can further enhance performance.

High-precision pressure-based metering requires predictive models of pressure drop along the flow path. Related dispensing studies have quantified the influence of pressure parameters on deposited volume in industrial air dispensers and drop-on-demand inkjet systems [26,27]. Because fluid viscosity is strongly temperature-dependent, pressure losses can vary substantially with temperature. Models and simulations of in-syringe flow have quantified how needle temperature influences dispensing error [28]. Related studies have used heating to modulate microfluidic pressure [29] and to stabilize pressure fluctuations [30], but the explicit use of thermal management to enable precision metering remains limited. Prior work shows good agreement between theoretical hydraulic resistance, computed from channel geometry under the Hagen–Poiseuille assumption, and experimental measurements. However, at very low flow rates, deviations increase, and the results become sensitive to hydrostatic head variations in the setup as well as to sensor dependence on temperature and fluid properties [31]. In the present system, the transfer line has an inner diameter no greater than one millimeter and a length of at least one meter, which further increases sensitivity to axial temperature gradients and viscosity variations. At lower flow rates, residence time in the transfer line increases, amplifying the effect of axial temperature gradients in an unheated tube and increasing uncertainty in viscosity and pressure loss. At higher flow rates, residence time decreases and this effect is reduced, although the required pressure increases due to viscous losses.

Another flow feature relevant to improving agreement between physical models and experimental observations is apparent wall slip, which may be associated with gas microbubbles or nanobubbles on hydrophobic channel or tube walls. Due to slip, experimentally measured flow rates can exceed the no-slip Hagen–Poiseuille prediction. A canonical analysis for circular pipes demonstrated that, when slip (shear-free) patches are periodically distributed along the wall, pressure-driven Stokes flow admits an effective slip length that depends on the pattern geometry [32]. This phenomenon has been studied extensively, and in nano- and micro-flows, slip has been reported to depend on interfacial conditions such as surface wettability, charge, and roughness [33]. Recent work, in particular, reports that nanobubbles on hydrophobic Teflon-based surfaces can generate pronounced slip effects that are not captured by classical models [34]. Across diverse geometries, combined analytical and experimental studies show that deviations observed in real microchannels can be explained by imposing a slip boundary condition, and that smaller channels exhibit more dominant slip effects [35]. Comparative theory–simulation–experiment studies further indicate that, in the presence of surface nanobubbles, the slip length can increase by orders of magnitude, with its value governed by the interfacial state (e.g., contact angle, bubble height, and surface coverage) [36].

In this study, we develop and validate a pressure-driven liquid transfer system for laboratory automation, together with a physics-based model and a calibration method. While feedback control can improve robustness by rejecting disturbances and compensating for unmodeled dynamics [25,37], the present study focuses on model-based feedforward pressure commands computed from a calibrated pressure-driven flow model. The system routes fluid through a single PTFE transfer tube enclosed by a heated jacket to maintain near-isothermal transport, thereby reducing temperature-driven variations in viscosity and pressure loss during dispensing.

The main contributions include a pressure-drop model that incorporates temperature-dependent viscosity and PTFE thermal expansion, as well as an identification procedure that captures systematic deviations from the no-slip prediction in submillimeter tubing using an effective slip length obtained through linear regression. The resulting pressure command can then be computed for a target flow rate at a specified temperature.

The remainder of this paper is organized as follows. Section 2 introduces the laboratory automation platform concept and the performance evaluation testbed. Section 3 derives the theoretical flow model and outlines operating strategies for accurate dispensing. Section 4 presents and analyzes the experimental results. Section 5 concludes with key findings and directions for future work.

## 2. System Design for Laboratory Automation

### 2.1. Device Architecture

**Figure 1:**
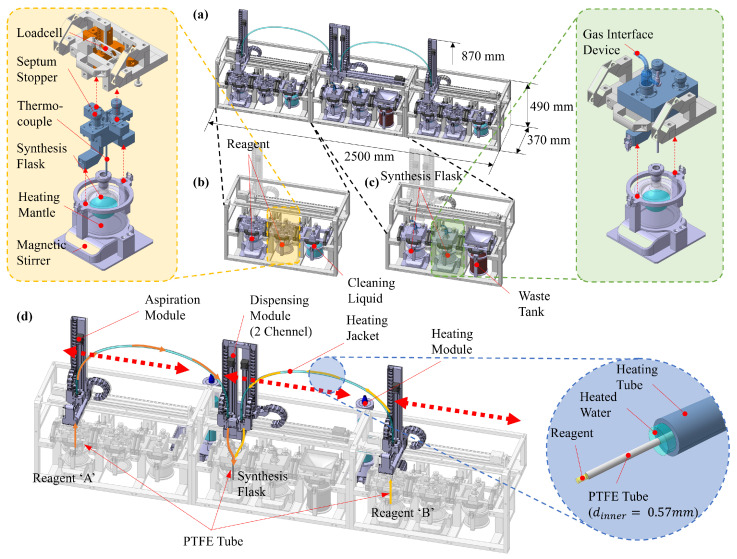
(**a**) Concept design of laboratory automation device, (**b**) reagent storage unit, (**c**) multi-step synthesis unit, (**d**) pressure-driven precise liquid transfer unit.

The demand for laboratory automation is increasing, driven by the need for improved reproducibility, safety, and efficiency in chemical synthesis. The platform developed in this study is designed to address these requirements and comprises three primary units, as shown in Figure 1a: a Reagent Storage Unit (RSU), a Multi-step Synthesis Unit (MSU), and a pressure-driven Precise Liquid Transfer Unit (PLTU) that interconnects them. The RSU and MSU adopt a compact, modular, slot-based architecture suitable for installation within a standard fume hood, enabling flexible deployment across a wide range of experimental protocols.

The RSU stores and manages multiple reagents, as illustrated in Figure 1b. Each slot accommodates a module tailored to a specific container type. Optional attachments, such as a heating mantle or a magnetic stirrer, are available to maintain the required temperature and reagent homogeneity. An adjustable weighing frame equipped with a precision load cell monitors the remaining mass in real time and provides a reference for verifying the transferred volume.

The MSU serves as the reaction workspace and can accommodate reaction flasks of various sizes, as shown in Figure 1c. It provides precise control of reaction temperature and stirring rate. In addition, the unit incorporates a port for a gas-interface device, enabling inert-gas purging or vacuum operation and supporting the automated execution of syntheses that are sensitive to air and moisture.

The PLTU is responsible for the precise transfer of reagents from the RSU to a reaction flask mounted on the MSU, as shown in Figure 1d. This unit comprises three modules: an aspiration module positioned above the selected reagent in the RSU, a dispense module positioned above the target flask in the MSU, and a temperature-control module that regulates the temperature of the PTFE transfer tubing connecting the two. The aspiration and dispense modules are positioned by single-axis linear stages to reach the pickup location and target flask, respectively. The temperature-control module circulates a high-temperature heat-transfer fluid around the exterior of the PTFE tubing to minimize thermal losses, thereby enabling near-isothermal transfer. Because reagent viscosity is strongly temperature-dependent, the viscous pressure drop within the PTFE tubing varies significantly with temperature. For certain reagents, maintaining a fixed temperature setpoint is essential; therefore, minimizing modeling error in the temperature-specific pressure-loss calculation is critical for accurate delivery.

Within this modular platform, overall process reliability depends on quantitative and time-accurate liquid transfer. Stable reagent storage and well-controlled reaction conditions alone are insufficient if prescribed reagent volumes cannot be delivered with precise timing. Consequently, accurate, repeatable, and reliable dispensing is a cornerstone capability of the platform. This study focuses on the pressure-driven PLTU, develops a physics-based model describing pressure–flow–temperature coupling, and experimentally validates the unit’s metering performance.

### 2.2. Liquid-Transfer Testbed

**Figure 2:**
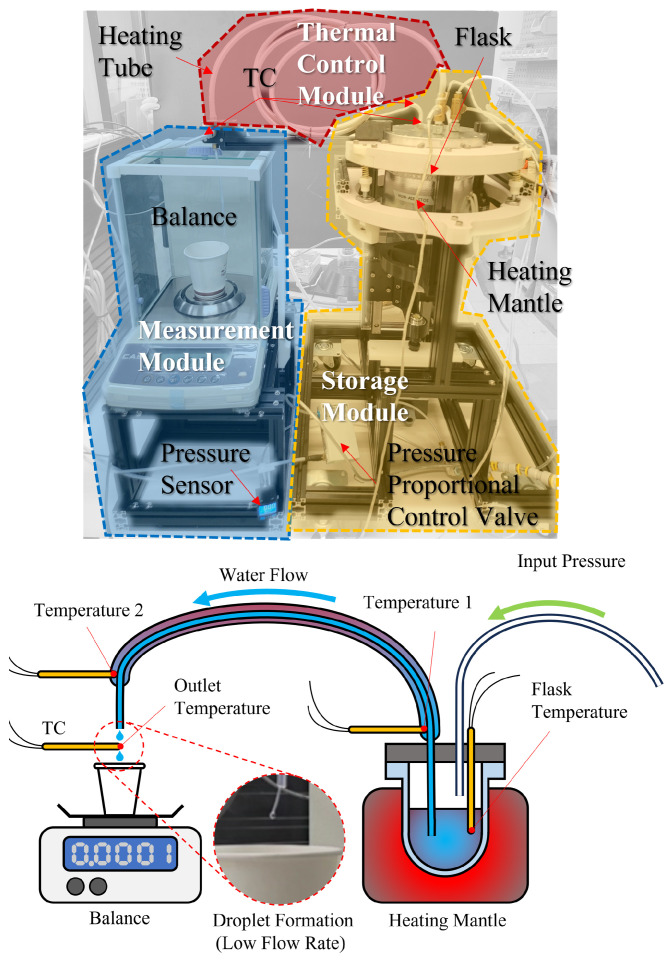
Validation testbed showing the storage module, thermal-control module, and measurement module.

A dedicated testbed was developed to evaluate pressure-driven liquid transfer under controlled thermal conditions. As shown in Figure 2, it integrates three modules: a measurement module for mass monitoring, a storage module with pressure and temperature control, and a thermal-control module that maintains a near-isothermal environment along the transfer tubing.

The storage module maintains a sealed headspace to exclude ambient air and to enable pressure-driven dispensing. Headspace pressure is regulated by a proportional control valve and monitored using an in-line pressure sensor. A single, continuous length of PTFE tubing—terminated in a pickup tube that extends near the bottom of the flask and connected via a push-to-connect bulkhead fitting—remains fully submerged in the liquid. This configuration ensures that positive headspace pressure drives discharge without entraining air.

The thermal-control module surrounds the PTFE tubing with a fluid jacket supplied by a recirculating bath (Jeiotech RW-0525G, JeioTech, Daejeon, Republic of Korea). Under near-isothermal conditions, fluid viscosity and density remain stable, thereby reducing discrepancies between theoretical and actual frictional pressure losses. In the absence of the heated jacket, heat loss to the ambient environment introduces temperature gradients that depend on flow rate, initial flask temperature, and dispensing duration. These gradients increase variability in viscosity and reduce the accuracy of pressure-loss modeling.

The measurement module consists of a laboratory balance (CAS CAUW-120D, CAS, Yangju, Republic of Korea) that provides real-time mass measurements. Thermocouples are installed on the reagent flask, at the inlet and outlet of the heated tube, and at a location in direct contact with the dispensed liquid. In-line pressure, flask temperature, jacket-fluid temperature, and outlet-fluid temperature are acquired and time-synchronized using a programmable logic controller (OMRON NX102-1200, OMRON, Kyoto, Japan). These synchronized measurements are used to assess the effectiveness of the thermal-control and mass-measurement subsystems and to characterize dispensing performance across reagents over a range of pressures and temperatures.

## 3. Pressure-Driven Flow Model

### 3.1. Thermal- and Slip-Augmented Model

Consider steady, incompressible, laminar, Newtonian flow through a straight circular tube with inlet and outlet located at positions 1 and 2, respectively. We assume a fully developed velocity profile, negligible elevation difference, and negligible kinetic energy change between the inlet and outlet. Under isothermal conditions and assuming no slip at the wall, the gauge pressure $P_{g}$ required to achieve a target volumetric flow rate $Q_{t}$ follows the Hagen–Poiseuille relation:

(1) $$P_{1}+\rho gz_{1}+\frac{1}{2}\rho v_{1}^{2}=P_{2}+\rho gz_{2}+\frac{1}{2}\rho v_{2}^{2}+\mu \left(T\right)\;\frac{8LQ_{t}}{\pi r^{4}},$$

which reduces to

(2) $$P_{g}=P_{1}-P_{2}=\mu \left(T\right)\;\frac{8LQ_{t}}{\pi r^{4}},$$

where μ(T) is the dynamic viscosity, *L* is the tube length, and *r* is the tube radius.

**Figure 3:**
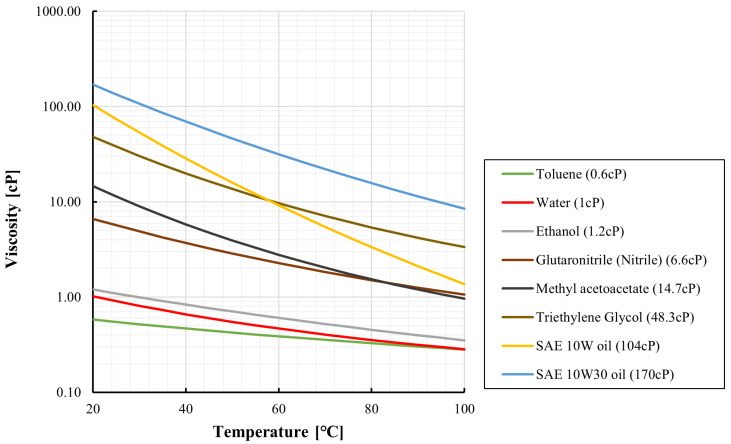
Temperature-dependent dynamic viscosity for representative liquids computed from the correlations.

Viscosity is strongly temperature-dependent. A commonly used correlation expresses μ as an exponential function of temperature [38]:

(3) $$\mu \left(T\right)=\mu_{20}\;\exp\;\left(C\left(\frac{T_{20}}{T}-1\right)\right),$$

where $\mu_{20}$ is the viscosity at 20  °C, *C* is an empirical constant, and $T_{20}$ and *T* are absolute temperatures. Over the temperature range from 273K to 373K, this correlation yields an accuracy of approximately 6%. Figure 3 shows temperature-dependent viscosities of ethanol, SAE 10W oil, and SAE 10W–30 oil computed from Equation (3). For water, a separate correlation with reported error within ±1% is also plotted in Figure 3 [38].

An alternative correlation for water is

(4) $$\mu \left(T\right)=\mu_{0}\;\exp\;\left(a+b\frac{T_{0}}{T}+c{\left(\frac{T_{0}}{T}\right)}^{2}\right),$$

where $\mu_{0}$ is the viscosity at 0 °C and *a*–*c* are regression parameters. For a range of organic liquids, a polynomial-in-*T* correlation provides approximately ±5% accuracy [39]:

(5) $$\mu \left(T\right)=10^{\alpha },\;\alpha =a+\frac{b}{T}+cT+dT^{2},$$

where *a*–*d* are regression coefficients.

Thermal expansion of PTFE alters both the tube length *L* and radius *r* as temperature varies. Using a linear thermal expansion coefficient α and reference values $L_{25}$ and $r_{25}$ at 25 °C, the pressure relation accounting for thermal expansion becomes

(6) $$P_{g}=\mu \left(T\right)\;\frac{8L_{25}Q_{t}}{\pi r_{25}^{4}{\left(1+\alpha \Delta T\right)}^{3}},$$

where ΔT=T−25 °C. Over the temperature interval considered here, the net effect corresponds to an expected increase of approximately 3% in flow rate at constant $P_{g}$.

**Figure 4:**
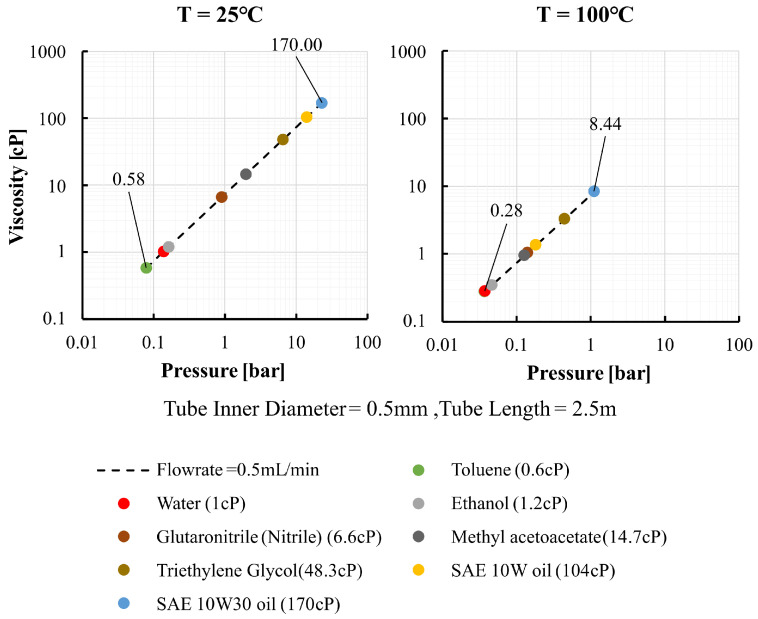
Computed required gauge pressure versus temperature for representative liquids.

Figure 4 shows the computed gauge pressure required to deliver $Q_{t}=0.5\;\mathrm{mL}\;\min^{-1}$ as a function of temperature for eight representative fluids. As temperature increases, viscosity decreases and the tube radius increases slightly due to thermal expansion, resulting in a reduced pressure requirement, often falling below 1bar at 100 °C. For example, the viscosity of SAE 10W–30 decreases from approximately 170cP at 25 °C to about 8.4cP at 100 °C, reducing the required pressure from 23.1bar to 1.1bar at the target flow rate.

The experimental validation reported in Section 4 is conducted using water. Figure 3 and Figure 4 are included solely as illustrative calculations to demonstrate how strongly viscosity and the required pressure can vary with temperature for representative liquids. These figures do not present experimental results from this study. They are intended to motivate the thermal-management design and to support interpretation of pressure-based metering when viscosity is temperature-sensitive.

Equations (1)–(6) assume no slip at the tube wall. In tubes with diameters below 1mm, gas microbubbles adhering to the surface can induce apparent slip at the fluid–wall interface [32]. As a result, the measured flow rate may exceed the no-slip Hagen–Poiseuille prediction. An effective slip length λ modifies the flow relation as

(7) $$Q_{s}=Q_{t}\;\left(1+\frac{4\lambda }{r}\right),$$

and the corresponding pressure requirement can be expressed using an experimentally identified effective slip length $\lambda_{\mathrm{update}}$ as

(8) $$P_{g}=\mu \left(T\right)\;\frac{8L_{25}Q_{s}}{\pi r_{25}^{3}{\left(1+\alpha \Delta T\right)}^{3}\left(r_{25}+4\lambda_{\mathrm{update}}\right)}.$$

Here, $\lambda_{\mathrm{update}}$ captures surface-condition and operating-state effects and is identified through preliminary calibration.

### 3.2. Slip-Length Identification by Linear Regression

The effective slip length $\lambda_{\mathrm{update}}$ is identified from preliminary experiments conducted over several pressure levels. In each run, the input pressure and output flow rate are measured, and the delivered volume is computed as a function of time. Because apparent slip scales the ideal flow by an approximately constant factor under a given operating condition, $\lambda_{\mathrm{update}}$ is estimated using ordinary linear regression between the target and measured volumes.

Multiplying Equation (7) by time *t* yields a linear relationship between the target volume $V_{t}=Q_{t}t$ and the measured volume $V_{s}=Q_{s}t$:

(9) $$V_{s}=V_{t}\;\left(1+\frac{4\lambda }{r}\right)\equiv a_{\lambda }V_{t},$$

where the slope $a_{\lambda }$ represents the slip-induced gain. To account for imperfect initialization and unmodeled transient effects, an intercept term is included:

(10) $$V_{s,k}=a_{0}+a_{\lambda }V_{t,k}+e_{k},\;k=1,2,\ldots ,n,$$

where samples are taken at uniform time intervals. In matrix form, this relation can be written as

(11) y=Xa+e,

with $y={\left[V_{s,1}\;\cdots \;V_{s,n}\right]}^{T}$, $a={\left[a_{0}\;a_{\lambda }\right]}^{T}$, and

$$X=\left[\begin{array}{cc} 1 & V_{t,1}\\ \vdots & \vdots \\ 1 & V_{t,n} \end{array}\right].$$

The least-squares estimate minimizes ${∥e∥}_{2}^{2}$ and satisfies the normal equations

(12) $$X^{T}X\;\hat{a}=X^{T}y.$$

Given the fitted slope ${\hat{a}}_{\lambda }$, the effective slip length is computed as

(13) $$\lambda =\frac{\left({\hat{a}}_{\lambda }-1\right)\;r_{\mathrm{eff}}}{4},\;r_{\mathrm{eff}}=r_{25}\;\left(1+\alpha \Delta T\right),$$

which is consistent with the thermally expanded radius used in Section 3.1. Substituting Equation (13) into Equation (8) yields

(14) $$P_{g}=\mu \left(T\right)\;\frac{8L_{25}Q_{s}}{\pi r_{25}^{4}{\left(1+\alpha \Delta T\right)}^{3}\;{\hat{a}}_{\lambda }},$$

so that the required gauge pressure is corrected by the experimentally identified gain ${\hat{a}}_{\lambda }$, which captures the net deviation from the ideal no-slip prediction under the tested configuration. In this work, λ is treated as an effective calibration parameter, and its identified value can depend on the reagent properties and the assembled flow path, including the tube inner-surface condition and fittings. Consequently, changes in the reagent or in the tube and fitting condition may require re-identification of λ to maintain accuracy in the open-loop compensation approach, while the model structure in Equations (7)–(14) can be retained.

## 4. Experimental Results and Discussion

### 4.1. Dispensing Without Heating Tube

Baseline dispensing experiments were conducted without heating the transfer tube. Water was used as the working fluid. The supply pressure was incremented from 0.1 to 0.9bar in steps of 0.1bar, while the dispensed mass was recorded continuously. Because the measurements included transient responses during pressure changes, steady-state segments were extracted, and the flow rate was estimated as the ordinary least-squares slope of mass versus time for each segment.

For each run, the internal flask temperature and the outlet-fluid temperature were logged in real time. Viscosity was computed from Equation (4) using the average internal flask temperature. The theoretical target flow rate was obtained from Equation (15), which is derived from Equation (6). Thermal expansion of PTFE was neglected at room temperature (RT) and included at elevated temperatures using the reference defined in Section 3.1. The experimental conditions and modeling parameters are summarized in Table 1. The relative flow error is defined as

$$\varepsilon_{Q}=\frac{Q_{\mathrm{meas}}-Q_{t}}{Q_{t}}\times 100\%.$$

(15) $$Q_{t}=\frac{\pi r_{25}^{4}{\left(1+\alpha \Delta T\right)}^{3}P_{g}}{8L_{25}\;\mu \left(T\right)}.$$

**Figure 5:**
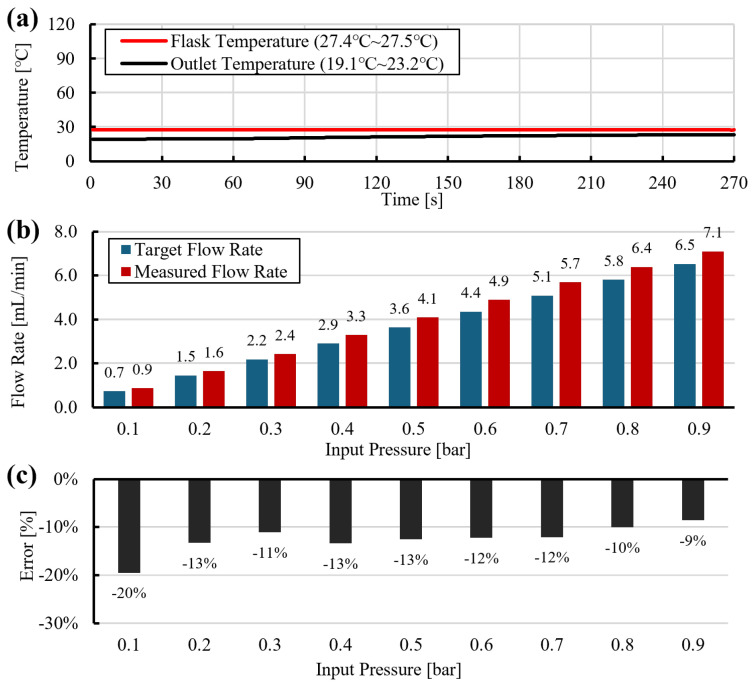
Dispensing without heated tube at room temperature (RT): (**a**) temperature traces, (**b**) pressure profile, (**c**) measured flow rate and model prediction using Equation (15).

At RT, the measured flow rates were generally 1–12% higher than the model prediction (Figure 5c). As shown in Figure 5a, the outlet temperature was slightly lower than the internal flask temperature, and viscosity was evaluated using the internal temperature, which marginally underestimates μ(T). The resulting positive error therefore suggests the presence of apparent slip along the 1mm diameter tube, leading to higher flow rates than predicted by the no-slip model.

**Figure 6:**
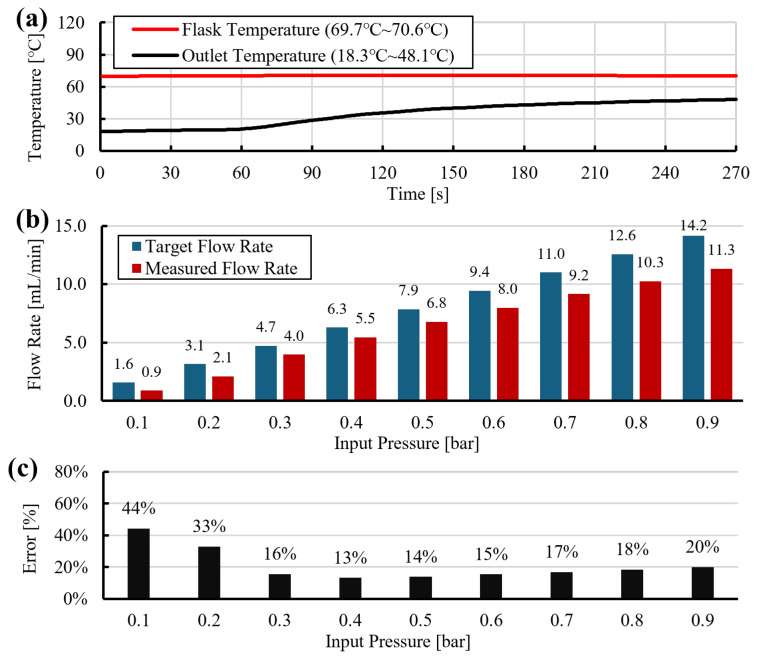
Dispensing without heated tube at 70 °C: (**a**) temperature traces, (**b**) pressure profile, (**c**) measured flow rate and model prediction using Equation (15).

At 70 °C, the measured flow rates were 12–44% lower than the prediction (Figure 6b,c). This discrepancy arises because the unjacketed PTFE tube loses heat to the ambient environment. As a result, the outlet temperature is lower than the initial flask temperature and increases gradually as pressure and flow rate rise (Figure 6a). These temperature gradients cause viscosity to vary along the tube and over time, violating the isothermal assumption and leading to underestimation of the pressure drop in Equation (15).

### 4.2. Dispensing Experiments with Heated Tube

**Figure 7:**
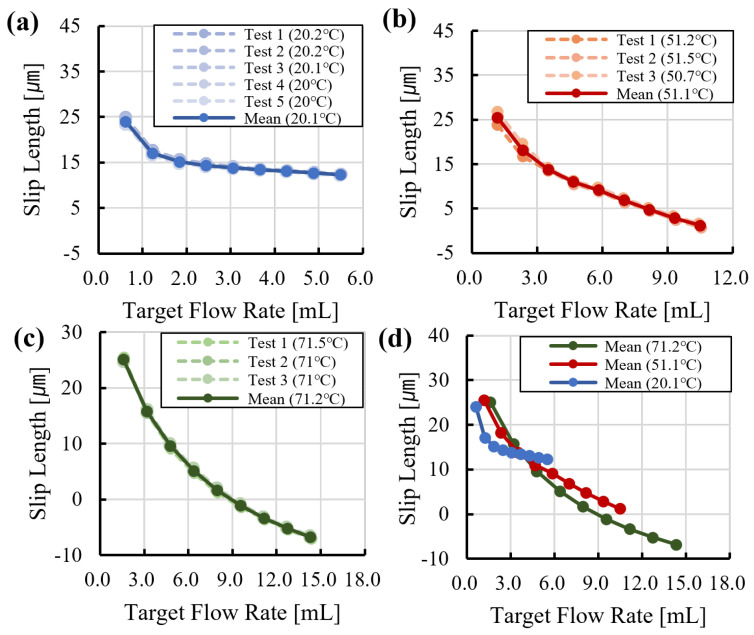
Estimated effective slip length as a function of flow rate and temperature: (**a**) 20 °C, (**b**) 50 °C, (**c**) 70 °C, (**d**) trend versus flow rate.

The heated tube module reduces heat loss from the transfer line and maintains near-isothermal conditions during liquid transport. Under stable temperature conditions, viscosity remains approximately constant, which improves agreement between the pressure-drop model and experimental measurements. Residual deviation from the no-slip prediction can still occur due to wall-related effects and may include gas bubbles at the interface [34]. In this work, the microscopic origin of these deviations was not directly measured, and the identified parameter is used as an effective correction that accounts for wall-related effects and the assembled flow path. The effective slip length was identified using the regression procedure described in Equations (9)–(13), and the results are summarized in Figure 7.

As shown in Figure 7a–c, the estimated slip length is generally consistent across temperatures, with representative values of 1.6 μm at 20 °C, 3.0 μm at 50 °C, and 0.9 μm at 70 °C. Figure 7d shows that the estimated slip length is larger at lower pressure and flow-rate conditions and decreases as the flow rate increases. This trend is consistent with a reduction in the identified effective slip parameter at higher flow rates.

**Figure 8:**
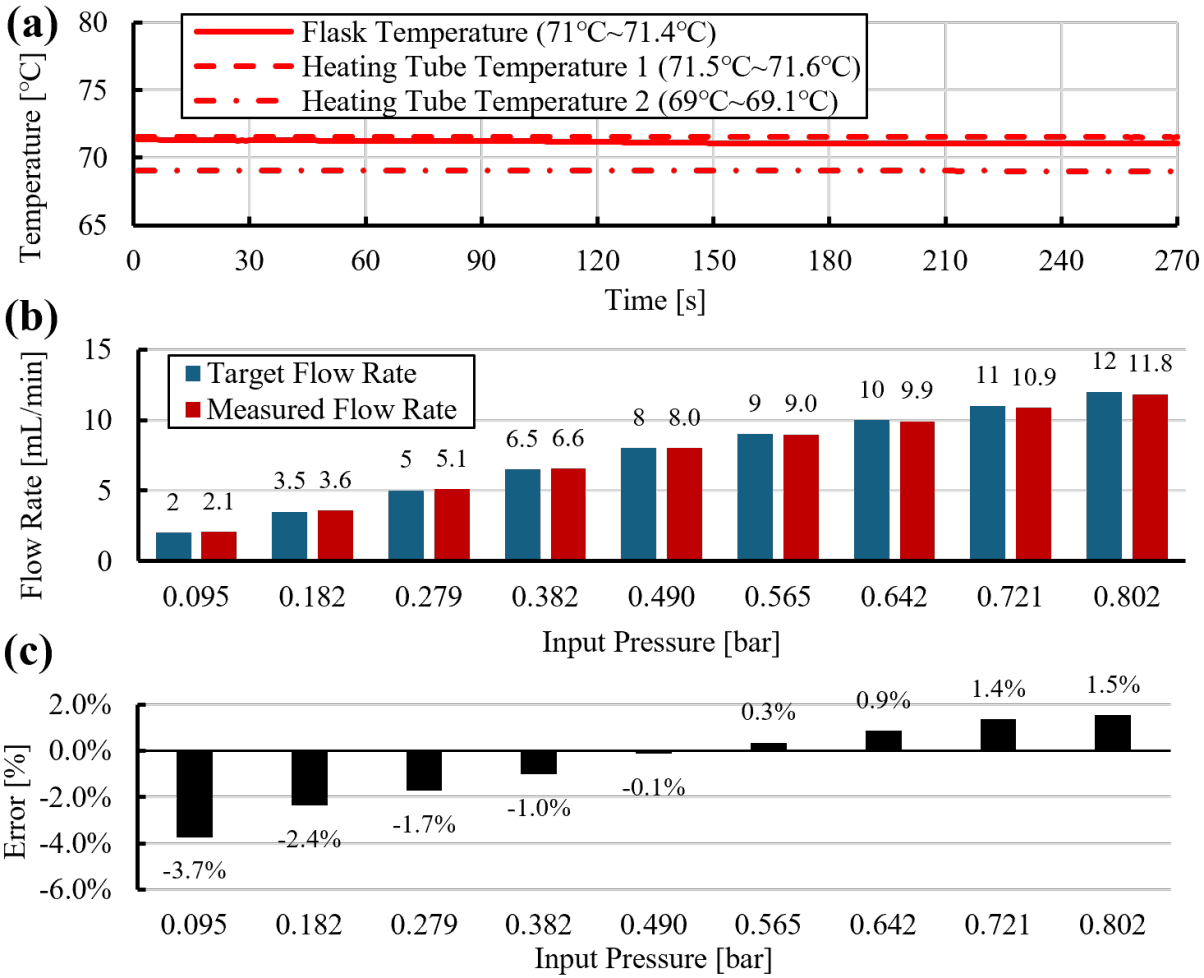
Dispensing at 70 °C with heated tube under slip compensation: (**a**) temperature history, (**b**) target and measured flow rates versus input pressure, (**c**) relative flow error versus input pressure.

In Figure 7c, the identified parameter λ assumes negative values at higher pressures. This occurs when the measured flow rate is lower than the no-slip Hagen–Poiseuille prediction for an ideal straight tube. In the present setup, this behavior suggests that additional pressure losses in the testbed, such as those arising from fittings and other minor losses, are not captured by the straight-tube model. Accordingly, λ is treated as an effective calibration parameter that represents the net deviation from the ideal prediction under the assembled flow path. With preliminary calibration, the compensated model achieves accurate dispensing, as demonstrated in Figure 8.

**Figure 9:**
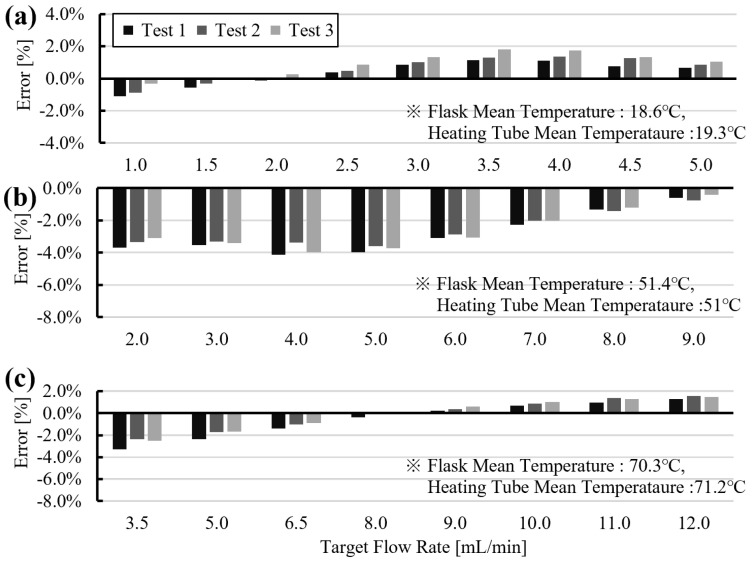
Tracking results using slip-compensated pressure commands. Relative flow error versus target flow rate at (**a**) 18.6 °C, (**b**) 51.4 °C, and (**c**) 70.3 °C.

Based on the preliminary experiments, slip-length values were mapped as a function of target flow rate (Figure 7). A polynomial fit was then used to describe slip length over the tested flow-rate range. Substituting the fitted slip length and the target flow rate into Equation (14) yielded the required gauge pressure. Using this model-based compensation, dispensing accuracy within ±4% of the target flow rate was achieved, as shown in Figure 9.

As shown in Figure 9a, at room temperature, the dispensing system applied an appropriate slip-compensated pressure, resulting in a flow-rate deviation of less than 2%. However, in experiments conducted at approximately 50 °C (Figure 9b) and 70 °C (Figure 9c), a deviation of approximately 2–4% was observed in the low-flow regime. This behavior is attributed to slip effects associated with microbubbles, which become more pronounced and exhibit higher sensitivity as the flow rate decreases and the temperature increases. This trend is consistent with Figure 5c and Figure 6c, where flow-rate deviation increases at lower flow rates at both room temperature and 70 °C, and with Figure 7, which shows a steep slope in the effective slip-length curve in the low-flow region.

## 5. Conclusions

This work presented a pressure-driven liquid transfer system for laboratory automation, together with an analytical model and an experimental testbed for validation. The system routes fluid through a single PTFE tube and maintains near-isothermal transport conditions, thereby reducing discrepancies between predicted and measured pressure drops. The model incorporates viscosity–temperature dependence and PTFE thermal expansion, whereas apparent wall slip in submillimeter tubing is represented by an effective slip length identified through linear regression.

With the heated tube and slip-compensated pressure commands derived from the model, the system achieved dispensing accuracy within ±4% over the tested operating range. Compared with the unheated configuration, which exhibited temperature gradients along the transfer line, the near-isothermal setup stabilized viscosity and improved agreement between experimental measurements and theoretical predictions.

The main limitations of the approach include sensitivity to tube surface condition and diameter uniformity, as well as the requirement for preliminary calibration for each reagent. Experimental validation in this study was performed using water, and extension to other reagents will require additional calibration and validation under the corresponding temperature conditions. Long-term durability was not evaluated in this study, and system reliability under extended operation should be assessed, with particular attention to time-dependent deformation and sealing performance at fittings subjected to repeated thermal exposure and sustained pressure.

Future work will extend the range of reagents and investigate methods to reduce sensitivity to disturbances and model mismatch. One practical direction is the implementation of closed-loop pressure regulation using a proportional valve and pressure sensing, with mass-based metering feedback when a balance is available. Disturbance-rejection strategies may also be explored to improve robustness under unmodeled losses and variations in operating conditions.

In addition, we will evaluate the sensitivity of the identified effective calibration parameter to tube inner-surface modifications commonly used in millifluidic applications and assess whether the same calibration framework remains valid for processed surface conditions. Finally, we plan to generalize the approach to multi-precursor recipes and time-varying flow profiles within the same modular platform.

## Figures and Tables

**Table 1:** System parameters used for modeling and analysis.

Parameter	Test	Symbol	Value	Unit
Tube radius (at 25 °C)		$r_{25}$	0.285	mm
Tube length (at 25 °C)		$L_{25}$	2.5	m
Viscosity	Test 1	μ(300.64K)	$8.57\times 10^{-4}$	$\mathrm{kg}\;m^{-1}\;s^{-1}$
	Test 2	μ(300.54K)	$8.59\times 10^{-4}$	
	Test 3	μ(300.39K)	$8.62\times 10^{-4}$	
	Test 4	μ(343.52K)	$4.02\times 10^{-4}$	
Thermal expansion coefficient		α	$1.24\times 10^{-4}$	C−1∘

## Data Availability

The data that support the findings of this study are available on request from the authors.
